# Efficacy of an Artificial Intelligence App (Aysa) in Dermatological Diagnosis: Cross-Sectional Analysis

**DOI:** 10.2196/48811

**Published:** 2024-07-02

**Authors:** Shiva Shankar Marri, Warood Albadri, Mohammed Salman Hyder, Ajit B Janagond, Arun C Inamadar

**Affiliations:** 1 Department of Dermatology, Venereology and Leprosy Shri B M Patil Medical College, Hospital and Research Centre BLDE (Deemed to be) University Vijayapura, Karnataka India

**Keywords:** artificial intelligence, AI, AI-aided diagnosis, dermatology, mobile app, application, neural network, machine learning, dermatological, skin, computer-aided diagnosis, diagnostic, imaging, lesion

## Abstract

**Background:**

Dermatology is an ideal specialty for artificial intelligence (AI)–driven image recognition to improve diagnostic accuracy and patient care. Lack of dermatologists in many parts of the world and the high frequency of cutaneous disorders and malignancies highlight the increasing need for AI-aided diagnosis. Although AI-based applications for the identification of dermatological conditions are widely available, research assessing their reliability and accuracy is lacking.

**Objective:**

The aim of this study was to analyze the efficacy of the Aysa AI app as a preliminary diagnostic tool for various dermatological conditions in a semiurban town in India.

**Methods:**

This observational cross-sectional study included patients over the age of 2 years who visited the dermatology clinic. Images of lesions from individuals with various skin disorders were uploaded to the app after obtaining informed consent. The app was used to make a patient profile, identify lesion morphology, plot the location on a human model, and answer questions regarding duration and symptoms. The app presented eight differential diagnoses, which were compared with the clinical diagnosis. The model’s performance was evaluated using sensitivity, specificity, accuracy, positive predictive value, negative predictive value, and *F*_1_-score. Comparison of categorical variables was performed with the *χ*^2^ test and statistical significance was considered at *P*<.05.

**Results:**

A total of 700 patients were part of the study. A wide variety of skin conditions were grouped into 12 categories. The AI model had a mean top-1 sensitivity of 71% (95% CI 61.5%-74.3%), top-3 sensitivity of 86.1% (95% CI 83.4%-88.6%), and all-8 sensitivity of 95.1% (95% CI 93.3%-96.6%). The top-1 sensitivities for diagnosis of skin infestations, disorders of keratinization, other inflammatory conditions, and bacterial infections were 85.7%, 85.7%, 82.7%, and 81.8%, respectively. In the case of photodermatoses and malignant tumors, the top-1 sensitivities were 33.3% and 10%, respectively. Each category had a strong correlation between the clinical diagnosis and the probable diagnoses (*P*<.001).

**Conclusions:**

The Aysa app showed promising results in identifying most dermatoses.

## Introduction

### Background

Diagnostic and therapeutic decisions in dermatology are heavily influenced by the morphology of diverse skin lesions. Traditionally, dermatological diagnoses are established by integrating the patient’s medical history, clinical examination, and, in some instances, dermoscopic and histopathologic analyses [1]. As it is predominantly a morphological feature–dependent specialty, dermatology is a field best suited for incorporating artificial intelligence (AI) image detection and recognition capabilities for aided diagnosis [2-5].

Given the discrepancy in access to dermatologists around the world, it is extremely crucial to be able to address patients’ medical needs [6]. Less than 1 dermatologist is available for every 100,000 individuals in India, and the majority of these specialists work in urban areas [7,8]. The diversity of cutaneous disorders and their striking resemblance to each other make accurate and efficient diagnosis challenging for general physicians. A delayed diagnosis due to a lack of specialists might significantly impact the patient’s quality of life [9,10]. Moreover, the high frequency of complicated inflammatory skin illnesses and the rising incidence of skin cancer have contributed to a surge in demand for dermatologists that is anticipated to continue growing in the future. Considering the potential for future pandemics, the capacity to deliver high-quality care virtually will likely continue to play a significant role in medicine [6,11]. AI-driven image diagnosis may be the solution to resolving these issues, allowing general practitioners to accurately detect common dermatological disorders by feeding a clinical image to a smartphone app [7,12,13].

Several AI-based applications have been created to assist in interpreting clinical pictures for various skin disorders, which are available for general use. By using these applications to examine concerning lesions, users may be prompted to schedule a telemedicine consultation or visit a dermatologist in person [6]. Medical personnel should have a thorough understanding of the merits and limitations of AI to promote its safe and efficient implementation [3,14]. Some of its merits include automating redundant assignments, performing constrained tasks, addressing spectator dependability issues, and ability to think outside the box. Conversely, there are unresolved legal, ethical, privacy, and liability issues associated with AI, and the inability to understand the decision-making process (ie, the “blackbox” nature) may limit its acceptability [2].

Despite the abundance of AI-integrated health apps accessible to the general public, there is limited research on their reliability, precision, and safety [6,15,16].

### The Aysa AI App

Aysa is an AI-enabled symptom-checker app developed by VisualDx. Aysa combines a problem-oriented clinical search with a well-curated medical image database comprising more than 120,000 medical images pertaining to 200 skin conditions in all Fitzpatrick skin types, expert medical knowledge, and cutting-edge machine learning (ML) techniques. The app uses the in-device framework such as Apple’s CoreML in iOS to accelerate ML tasks. Aysa can modify its results based on a user’s medical history, further personalizing the experience for consumers. The Aysa app is commercially available for download on iOS and Android devices [17].

By analyzing clinical images, patient demographic details, skin type, the morphology of the lesions, and associated symptoms, the app provides probable diagnoses for skin conditions and gives a detailed overview of the condition along with the urgency of consultation. This enables the user to learn more about their skin issues and make informed decisions, although it is not intended for diagnostic purposes. Image recognition and analysis occur on the device itself using the in-device AI framework. However, there is a lack of information regarding the type of neural network the app uses. Privacy is ensured by encrypting images during transit, which are then discarded after analysis. Patient profiles, associated cases, and images are in complete control of the user [17].

Although the app is marketed as a symptom-checker app and not for diagnostic purposes, it is imperative to determine its accuracy and reliability, as the general public might be misled by the results.

### Objective

The aim of this study was to validate an AI-based app (Aysa) as a preliminary diagnostic tool for Asian users with Fitzpatrick skin types III-V living in a semiurban town in India seeking consultation in a tertiary-care hospital for common skin conditions such as dermatitis, disorders of keratinization, papulosquamous disorders, pigmentary disorders, photodermatoses, skin infections and infestations, tumors, and other inflammatory conditions.

## Methods

### Source of Data

This observational cross-sectional study included 700 participants older than 2 years who consulted the dermatology outpatient department of a tertiary-care facility [Shri B M Patil Medical College, Hospital and Research Centre, BLDE (Deemed to be) University, Vijayapura, Karnataka, India] for common skin conditions between January 2023 and March 2023. All included patients were of Asian ethnicity with Fitzpatrick skin types III-V and presented with various skin conditions, which were grouped into the categories listed in Table 1. Malignant tumors were histopathologically confirmed. Hair and nail disorders and bullous disorders were excluded as the app is not designed to identify these conditions. Patients who had received prior treatment for their conditions and those who refused to authorize the inclusion of their images for the study were excluded.

**Table 1:** Various skin conditions included in the study grouped into broad categories.

Clinical category	Clinical conditions
Bacterial infections	Cellulitis, folliculitis, impetigo
Benign tumors	Acrochordon, dermatosis papulosa nigra, nevus, pyogenic granuloma, seborrheic keratosis, syringoma
Dermatitis	Atopic dermatitis, dyshidrotic dermatitis, hand dermatitis, nummular dermatitis, pityriasis alba
Disorders of keratinization	Acanthosis nigricans, ichthyosis, keratosis pilaris
Fungal infections	Candidiasis, dermatophytosis, pityriasis versicolor
Malignant tumors	Basal cell carcinoma, cutaneous lymphoma, squamous cell carcinoma
Other inflammatory disorders	Acne keloidalis nuchae, acne vulgaris, granuloma annulare, insect bite reaction, spider bite reaction, urticaria, vasculitis
Papulosquamous disorders	Lichen planus, psoriasis
Photodermatoses	Favre-Racouchot syndrome, polymorphous light eruption
Pigmentary disorders	Café-au-lait macule, freckles, melasma, vitiligo
Skin infestations	Pediculosis, scabies
Viral infections	Hand, foot, and mouth disease; herpes simplex 1 and 2 infections; herpes zoster; molluscum contagiosum; varicella; warts

### Ethical Considerations

The study was conducted in accordance with the Declaration of Helsinki (as revised in 2013). The study was approved by the Institutional Ethics Committee of BLDE (Deemed to be University; IEC/No. 09/2021). Informed consent was obtained from all individual participants and data were anonymized. No compensation was provided for study participation.

### Methodology

**Figure 1:**
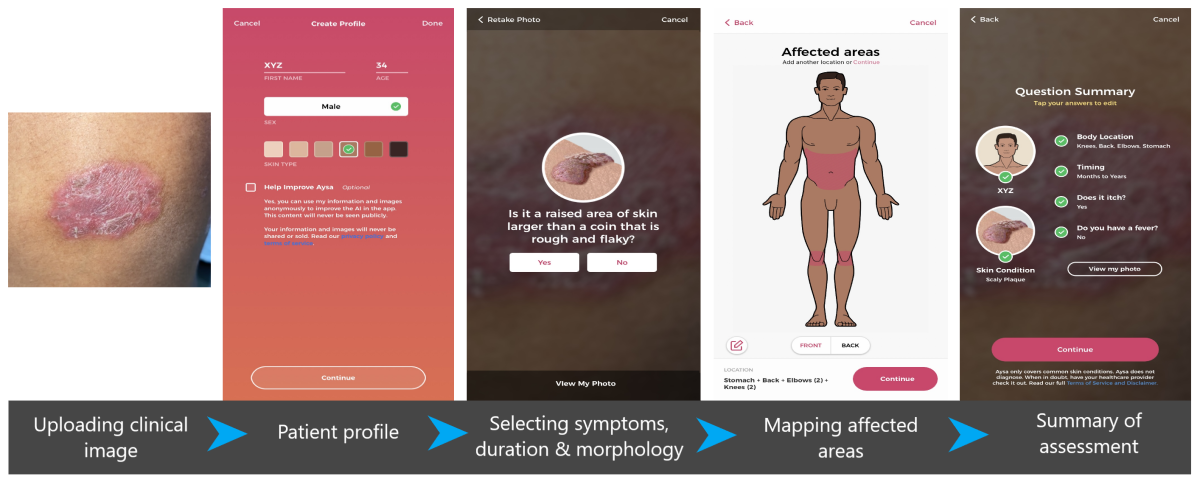
Images from the app depicting the workflow.

This manuscript has been prepared following the TRIPOD (Transparent Reporting of a multivariable prediction model for Individual Prognosis Or Diagnosis) checklist [18]. After detailed history and examination of the patients, the clinical diagnosis was established and verified by two expert dermatologists. Histopathological confirmation was obtained for suspicious lesions. Images of the skin lesions were captured on an iPhone 11 with a 12-megapixel sensor in a well-lit environment ensuring privacy. These images were then uploaded onto the Aysa app. A patient profile pertaining to age, sex, and skin type was created. Following this, the app identified the morphology of the skin lesions and ascertained the lesions by providing a description in colloquial language with pictorial representations. The location of the lesions was plotted on a human model put forward by the app, and certain questions relating to the duration of the skin lesions and associated symptoms were answered. Figure 1 provides images from the app depicting the workflow.

The app identifies 8 probable differential diagnoses for every skin condition. These were compared with the clinical diagnosis established by dermatologists.

### Statistical Analysis

Performance criteria such as sensitivity, specificity, positive predictive value (PPV), negative predictive value (NPV), accuracy, and *F*_1_-score were used to assess the model’s performance. Disease-specific sensitivity; specificity; PPV; NPV; accuracy; *F*_1_-score; and overall top-1, top-3, and all-8 sensitivities of the model were determined and represented as percentages with 95% CIs. The clinical diagnosis had to be predicted among the top one, top three, and all probable diagnoses to be eligible for top-1, top-3, and all-8 sensitivities, respectively. Data were analyzed using JMP Pro 16 software version 16 (SAS Institute). Categorical variables were compared with the χ^2^ test and statistical significance was considered at *P*<.05.

## Results

### Demographics and Basic Characteristics

This study involved a total of 700 patients. More than half the sample comprised male patients (n=418, 59.7%) and the greatest proportion of patients were in the age range of 10-19 years (n=178, 25.4%). Patients presented with a wide range of conditions, which were grouped into 12 categories: bacterial infections (n=22, 3.1%), benign tumors (n=40, 5.7%), dermatitis (n=55, 7.8%), disorders of keratinization (n=28, 4.0%), fungal infections (n=97, 13.8%), malignant tumors (n=20, 2.8%), other inflammatory disorders (n=110, 15.7%), papulosquamous disorders (n=70, 10.0%), photodermatoses (n=21, 3.0%), pigmentary disorders (n=101, 14.4%), skin infestations (n=28, 4.0%), and viral infections (n=108, 15.4%).

### Performance of the App

The AI model demonstrated an aggregate top-1 sensitivity of 71% (95% CI 61.5%-74.3%), top-3 sensitivity of 86.1% (95% CI 83.4%-88.6%), and all-8 sensitivity of 95.1% (95% CI 93.3%-96.6%). The top-1, top-3, and all-8 sensitivities; specificity; PPV; NPV; accuracy; and *F*_1_-score of the grouped skin conditions are provided in Table 2. The top-1 sensitivities of skin infestations, disorders of keratinization, other inflammatory conditions, and bacterial infections were 85.7%, 85.7%, 82.7%, and 81.8%, respectively. All the classes displayed high specificity, accuracy, and NPV. All categories showed a significant association between clinical and probable top-1, top-3, and all-8 diagnoses (*P*<.001).

**Figure 2:**
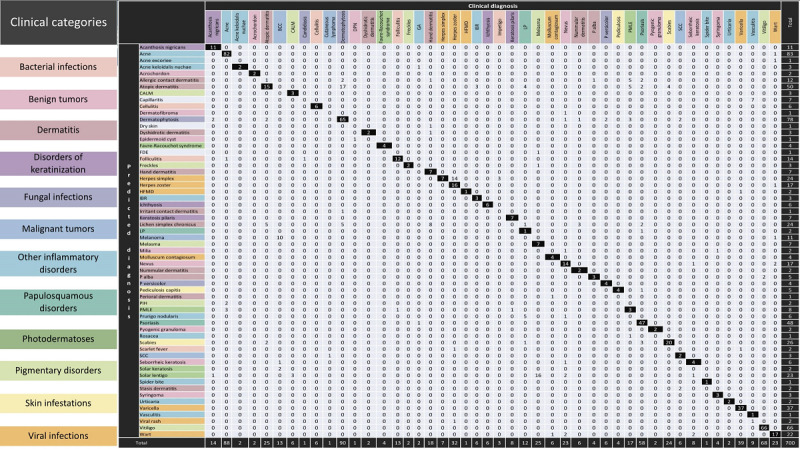
Confusion matrix between top-1 predicted and clinical diagnoses in individual skin conditions.

**Figure 3:**
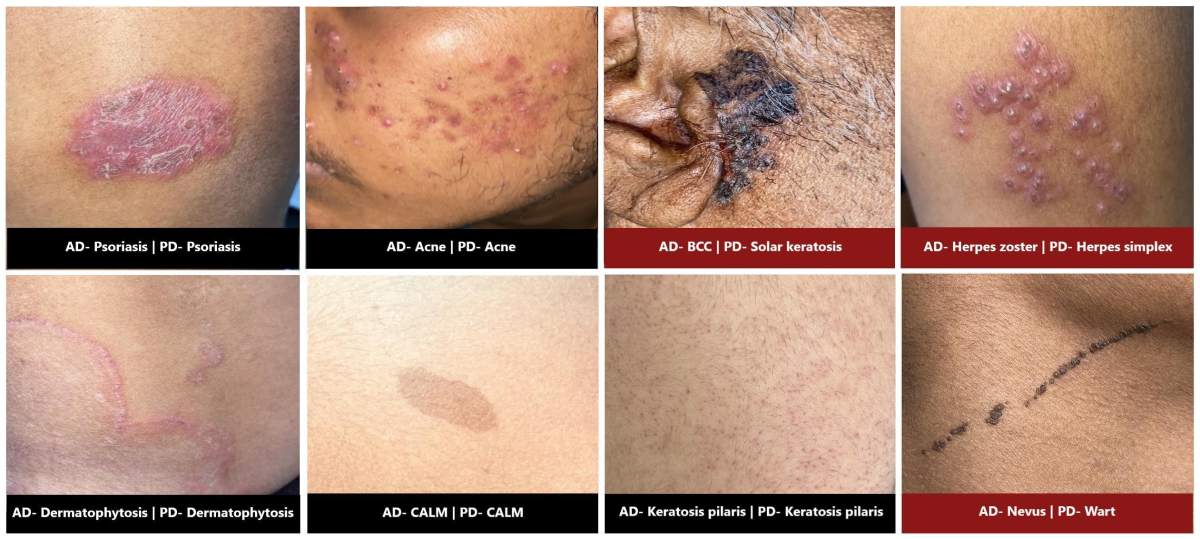
Clinical images with clinical and predicted diagnoses. AD: actual diagnosis; BCC: basal cell carcinoma: CALM: café-au-lait macule; PD: predicted diagnosis.

Table 3 shows the top-1, top-3, and all-8 sensitivities; specificity; PPV; NPV; accuracy; and *F*_1_-score of the most common individual skin conditions found among the broader categories. The top-1 sensitivities of acne, dermatophytosis, psoriasis, lichen planus, and vitiligo were 93.2%, 72.2%, 81%, 27.7%, and 97%, respectively. The confusion matrix between probable top-1 diagnoses and clinical diagnoses is illustrated in Figure 2. Figure 3 depicts representative clinical images with their corresponding clinical and predicted diagnoses.

**Table 2:** Performance metrics of the probable diagnoses of the app compared to clinical diagnoses grouped according to skin condition category (N=700).

Clinical category	Cases, n (%)	Sensitivity, % (95% CI)				Specificity, % (95% CI)		PPV^a^, % (95% CI)		NPV^b^, % (95% CI)		Accuracy, % (95% CI)		*F*_1_-score		*P* value
		Top-1	Top-3	All-8												
Bacterial infections	22 (3.1)	81.8 (59.7-94.8)	90.9 (70.8-98.9)	100 (84.6-100)	99.7 (98.9-99.9)		90 (68.9-97.3)		99.4 (98.6-99.8)		99.1 (98.1-99.7)		0.857		<.001	
Benign tumors	40 (5.7)	62.5 (45.8-77.3)	85 (91.2-100)	92.5 (79.6-98.4)	99.5 (98.7-99.9)		89.3 (72.4-96.3)		97.8 (96.7-98.5)		97.4 (95.9-98.5)		0.735		<.001	
Dermatitis	55 (7.8)	52.7 (38.8-66.3)	78.1 (64.9-88.1)	98.1(90.3-99.9)	94.3 (92.2-95.9)		43.9 (34.3-53.9)		95.9 (94.6-96.9)		91 (88.6-93)		0.479		<.001	
Disorders of keratinization	28 (4)	85.7 (67.3-95.9)	96.4 (81.6-99.9)	100 (87.7-100)	100 (99.4-100)		100		99.4 (98.5-99.8)		99.4 (98.5-99.8)		0.923		<.001	
Fungal infections	97 (13.8)	71.1 (61-79.9)	86.6 (78.2-92.7)	96.9 (91.2-99.4)	98.1 (96.8-99)		86.2 (77.5-91.9)		95.5 (93.9-96.7)		94.4 (92.5-96)		0.779		<.001	
Malignant tumors	20 (2.8)	10 (1.2-31.7)	10 (1.2-31.7)	25 (8.6-49.1)	99.8 (99.2-100)		66.7 (15.9-95.5)		97.4 (97-97.8)		97.3 (95.8-98.4)		0.173		<.001	
Other inflammatory conditions	110 (15.7)	82.7 (743-89.3)	95.4 (89.7-98.5)	100 (96.7-100)	82.7 (74.3-89.9)		91 (84-95.1)		96.8 (95.3-97.9)		96 (94.3-97.3)		0.866		<.001	
Papulosquamous disorders	70 (10)	68.6 (56.4-79.1)	80 (68.7-88.6)	98.6 (92.3-99.9)	99.8 (99.1-100)		97.9 (87-99.7)		96.6 (95.3-97.6)		96.7 (95.1-97.9)		0.806		<.001	
Photodermatoses	21 (3)	33.3 (14.6-56.9)	61.9 (38.4-81.9)	100 (83.9-100)	95.6 (93.7-97)		18.9 (10.4-31.9)		97.9 (97.1-98.4)		93.7 (91.6-95.4)		0.241		<.001	
Pigmentary disorders	101 (14.4)	77.2 (67.8-84.9)	97 (91.6-99.4)	97 (91.6-99.4)	99.7 (98.8-99.9)		97.5 (90.7-99.4)		96.3 (94.8-97.4)		96.4 (94.8-97.7)		0.861		<.001	
Skin infestations	28 (4)	85.7 (67.3-95.9)	100 (87.7-100)	100 (87.7-100)	98.9 (97.9-99.6)		77.4 (61.8-87.9)		99.4 (98.5-99.7)		98.4 (97.2-99)		0.813		<.001	
Viral infections	108 (15.4)	75.9 (66.7-83.6)	86.1 (78.1-92)	92.6 (85.9-96.7)	98.6 (97.3-99.4)		91.1 (83.6-95.3)		95.7 (94.1-96.9)		95.1 (93.3-96.6)		0.828		<.001	

^a^PPV: positive predictive value.

^b^NPV: negative predictive value.

**Table 3:** Performance metrics of the probable diagnoses of the app compared to clinical diagnoses for the most significant individual skin conditions (N=700).

Individual skin conditions	Cases, n (%)	Sensitivity, % (95% CI)			Specificity, % (95% CI)	PPV, % (95% CI)	NPV, % (95% CI)	Accuracy, % (95% CI)	*F*_1_-score	*P* value
		Top-1	Top-3	All-8						
Acne	88 (12.6)	93.2 (85.7-97.5)	100 (95.8-100)	100 (95.8-100)	99.8 (99-100)	98.8 (92-99.8)	99 (97.9-99.5)	99 (97.9-99.6)	0.959	<.001
Dermatophytosis	90 (12.9)	72.2 (61.8-81.1)	88.9 (80.5-94.5)	100 (95.9-100)	97.9 (96.4-98.9)	83.3 (74.2-89.7)	95.9 (94.5-97)	94.6 (92.6-96.1)	0.773	<.001
Psoriasis	58 (8.3)	81 (68.6-90.1)	91.4 (81-97.1)	100 (93.8-100)	99.8 (99.1-100)	97.9 (86.9-99.7)	98.3 (97.1-99)	98.3 (97-99.1)	0.886	<.001
Lichen planus	12 (1.7)	8.3 (0.2-38.5)	25 (5.5-57.1)	91.7 (61.5-99.8)	99.9 (99.1-100)	50 (6.2-93.8)	98.4 (98.1-98.7)	98.3 (97-99.1)	0.142	<.001
Vitiligo	68 (9.7)	97 (89.8-99.6)	100 (94.7-100)	100 (94.7-100)	100 (99.4-100)	100	99.68 (98.8-99.9)	99.7 (98.9-99.9)	0.985	<.001

## Discussion

### Key Findings

This study analyzed the diagnostic accuracy of a commercially available AI-based health care app for various skin conditions. The app uses ML to analyze the clinical images, predict the probable diagnoses, and provide personalized guidance to the user.

Most of the patients included in this study had inflammatory conditions, pigmentary disorders, and infectious diseases. The top-1, top-3, and all-8 sensitivities for the AI model were collectively 71% (95% CI 61.5%-74.3%), 86.1% (95% CI 83.4%-88.6%), and 95.1% (95% CI 93.3%-96.6%), respectively. The app demonstrated high sensitivities in most categories in top-1 probable diagnoses, except in benign tumors, dermatitis, malignant tumors, and photodermatoses. When the top-3 probable diagnoses were considered, the sensitivities increased in all the categories except malignant disorders. In the case of photodermatoses, the sensitivity increased from 33.3% to 61.9% and subsequently to 100% when top-3 and all-8 probable diagnoses were considered, respectively. However, in the case of malignant disorders, the sensitivity remained the same and only increased to 25% when all 8 probable diagnoses were taken into account.

When considering specific skin conditions, the app could diagnose acne, dermatophytosis, psoriasis, and vitiligo with good sensitivity. Among papulosquamous disorders, the top-1 sensitivities of psoriasis and lichen planus were 81% and 27.7%, respectively. Among other inflammatory disorders, the top-1 sensitivity of acne was 93.2%, which increased to 100% when top-3 diagnoses were included.

Examination of the confusion matrix showed that the number of false negatives for herpes zoster was equal to the number of true positives, with herpes simplex being the most predicted diagnosis among false negatives (predicted in 43.7% of all patients with herpes zoster). This can likely be attributed to the morphology and location of the lesions. Most basal cell carcinoma cases (76.9%) were predicted as melanoma in the top-1 diagnosis.

### Comparison With Similar Studies

We further sought to compare the diagnostic accuracy of the Aysa app with similar algorithms under comparable study conditions. However, direct comparison would only be possible if the same image sets were used in the evaluation of various algorithms.

Marri et al [2] assessed the Tibot AI app in diagnosing skin conditions in 600 patients. For the predicted top-3 diagnoses given by the app, the mean prediction accuracy was 96.1% (95% CI 94.3%-97.5%) and for the exact diagnosis it was 80.6% (95% CI 77.2%-83.7%).

Using clinical photos of skin lesions from patients with verified COVID-19, healthy individuals, and 18 common dermatoses, Mathur et al [19] developed a convolutional neural network (CNN)–based algorithm. The top-1 overall sensitivity for the diagnosis of 20 skin disorders was 87.65%, while the top-3 sensitivity was 96.72%.

Table 4 provides a comparison of the sensitivities, specificity, and PPV of AI algorithms of this study and the studies by Marri et al [2] and Mathur et al [19] in diagnosing various skin disorders. The sensitivity in the majority of the conditions was comparable in all the studies except for lichen planus and malignant tumors. Although the Tibot app evaluated by Marri et al [2] demonstrated higher sensitivity in diagnosing malignant tumors, it only gives a broad diagnosis, unlike the Aysa app, which predicts a specific diagnosis. In the study by Mathur et al [19], the CNN model predicted lichen planus with better sensitivity than achieved with the Aysa app.

Wu et al [20] evaluated the accuracy of a CNN model in diagnosing inflammatory skin conditions. The sensitivity and specificity of the model were found to be 94.4% and 97.2%, respectively, and the overall accuracy was 95.8%. For eczema and atopic dermatitis, the accuracy was 92.57%, with a sensitivity and specificity of 94.56% and 94.4%, respectively. The accuracy for psoriasis was 89.46%, with a sensitivity and specificity of 91.4% and 95.48%, respectively. In this study, the Aysa app showed an accuracy of 98.3% with a top-1 sensitivity of 81% and a specificity of 99.8% in the case of psoriasis. For atopic dermatitis, the accuracy was 91%, with a top-1 sensitivity and specificity of 52.7% and 94.3%, respectively.

Other studies have demonstrated the efficacy of AI in diagnosing benign and malignant dermatoses [21-23]. The performance of the CNN models evaluated by Esteva et al [22] and Han et al [23] was comparable to or better than the diagnostic ability of dermatologists. In this study, the Aysa app demonstrated a top-1 sensitivity of 62.5% and a specificity of 99.5% in identifying benign tumors. For malignant conditions, the top-1 sensitivity was 10% with a specificity of 99.8%.

**Table 4:** Comparison of the sensitivities, specificities, and positive predictive values (PPVs) of various artificial intelligence algorithms evaluated in this study and previous studies.

Skin conditions	This study								Marri et al [2]								Mathur et al [19]					
	Sensitivity, %			Specificity, %		PPV, %		Sensitivity, %				Specificity, %		PPV, %		Sensitivity, %				Specificity, %		PPV, %
	Top-1	Top-3					Top-1			Top-3					Top-1			Top-3				
Acne	93.2	100	99.8		98.8		92			99	99		91		92.3			97.9	99.1		91	
Bacterial infections	81.8	90.9	99.7		90		50			83	99		43		88.6^a^			95.3	99.2		89.6	
Benign tumors	62.5	85	99.5		89.3		71			100	98		69		—^b^			—	—		—	
Dermatitis	52.7	78.1	94.3		43.9		75			100	95		37		—			—	—		—	
Fungal infections	71.1	86.6	98.1		86.2		83			97	96		80		90^c^			98.3	97.9		89.2	
Lichen planus	8.3	25	99.9		50		—			—	—		—		81.2			96.2	99		84.7	
Malignant tumors	10	10	99.8		66.7		82			100	99		75		—			—	—		—	
Psoriasis	81	91.4	99.8		97.9		70			91	99		87		85.3			96.9	97.9		86	
Pigmentary disorders	77.2	97	99.7		97.5		89			99	99		96		—			—	—		—	
Skin infestations	85.7	100	98.9		77.4		69			94	99		75		—			—	—		—	
Viral infections	75.9	86.1	98.6		91.1		63			95	98		90		86.4^d^			95.3	99.4		85.2	

^a^Included impetigo and pyodermas only.

^b^These conditions were not included in the respective studies.

^c^Included tinea cruris, corporis, or faciei only.

^d^Included herpes zoster only.

### Implications

The Aysa app has proven to be effective in predicting most of the common dermatoses encountered in a population. In addition to skin analysis, the app provides in-depth details on the conditions in the form of an overview comprising the causes, symptoms, risk factors, course, prognosis, and treatment information; preconsultation advice; when to see a doctor; and differential diagnoses. Materials adapted from renowned textbooks, journal papers, PubMed, the World Health Organization, the Infectious Diseases Society of America, and the US Centers for Disease Control and Prevention are included in the content [17]. Notifying the patient of the urgency index is practical because skin disorders are typically ignored until they cause significant inconvenience. Thus, the Aysa app has the potential to motivate patients to seek medical care, improve patient engagement and participation, improve the efficiency and productivity of physicians, and reduce health care expenditure [24,25].

Health care practices can be enhanced by integrating advanced diagnostic knowledge using these AI-based health care systems. For a skin condition, images can be uploaded to a specialized dermatological AI system from a general practitioner’s office, and prompt analysis can be performed if the uploaded image is sufficient to reach a conclusion. This would help patients with low-risk conditions receive immediate reassurance about their concerns, while those with high-risk conditions can have a speedy referral to a specialist clinic [12]. Finding a balance that optimizes the advantages of AI while maintaining the humanistic touch is crucial for patient care.

### Limitations

Absence of image consistency in terms of focus, angle, and illumination is one of the main limitations of our study. Although the app can identify almost 200 skin disorders, this study included only 46 common conditions. The majority of the study population had infections, pigmentary disorders, and inflammatory illnesses. Photodermatoses and tumors were relatively less frequent in this population, which may account for the app’s poor performance in these categories. Additional research focusing on these conditions and others not included in this study may be required to validate the app’s performance. For simplicity of comprehension, specific skin conditions were categorized into broad groups. This could have given an impression of relatively consistent performance, as in the case of papulosquamous disorders, where the app showed good sensitivity to diagnose psoriasis but failed to diagnose lichen planus with the same sensitivity. Dermatological conditions have a diverse morphology based on various factors, including severity of the disease. This might hinder the ability of the app to provide an accurate diagnosis. Further studies correlating severity of the disease and other factors with the app’s diagnostic ability might be required.

There are certain drawbacks to the app. As it is designed for users above the age of 2 years, certain conditions such as infantile hemangioma, commonly encountered in clinical practice, could not be diagnosed. As the app is intended for assessing skin conditions, hair and nail disorders could not be included in the study. The preconsultation advice provided by the app contains information regarding over-the-counter medications appropriate for the condition. This may encourage the patient to self-medicate rather than seek consultation. Another limitation is the lack of transparency regarding the type of neural network used by the app despite our efforts to obtain that information.

### Conclusions

The Aysa app has demonstrated promising outcomes in the diagnosis of prevalent dermatological issues such as infections, inflammatory disorders, infestations, and pigmentary disorders. However, the app is unreliable at detecting photodermatoses and malignant tumors. Further improvement might be required for the app to be implemented in clinical practice.

## Appendix

Multimedia Appendix 1 TRIPOD checklist.

## Abbreviations

AI: artificial intelligence
CNN: convolutional neural network
ML: machine learning
NPV: negative predictive value
PPV: positive predictive value
TRIPOD: Transparent Reporting of a multivariable prediction model for Individual Prognosis Or Diagnosis

## Data Availability

The data sets generated and analyzed during this study are available from the corresponding author on reasonable request.
